# Malarial Haemoglobinuria*Read before Victoria Meeting, South Texas Medical Association, June 10, 1903.

**Published:** 1903-09

**Authors:** M. M. Pool

**Affiliations:** El Campo, Texas


### TEXAS MEDICAL JOURNAL.

ESTABLISHED JULY, 1885.

PUBLISHED MONTHLY.—SUBSCRIPTION $1.00 A YEAR.

VOL. XIX. AUSTIN, SEPTEMBER, 1903. No. 3.

### Original Contributions.

For Texas Medical Journal.

### Malarial Haemoglobinuria.*

BY M. M. POOL, M. D., EL CAMPO, TEXAS.

My reason for presenting this paper for your consideration is because the chairman of this section invited me to do so, and not because I have something new or original to engage your attention, for I am well aware that so much has been written upon this subject within the last few years, that all I may say concerning it will only be common knowledge to the medical profession of today; hence, I beg your indulgence while I rehearse that which has been gleaned from able authorities, and my own practical experience.

As the symptoms given by all writers upon this malady are practically the same, and no difficulty attends its diagnosis, I shall not bore you with rehearsals upon these parts of the subject, as they would doubtless be "chestnuts" to those scarcely attaining to mediocrity in the profession (there being none such in this association), but will consider its etiology and kidney pathology only to the extent necessary to elucidate my plan of treatment, the latter being the battle ground from whence I expect a volley of grapeshot from members of this association who diametrically oppose my views.

In the course of an intermittent or a remittent malarial fever,

*Read before Victoria Meeting, South Texas Medical Association, June 10, 1903.

2 Mj and often *without* the previous exhibition of quinine, a sudden disintegration of the red-blood corpuscles takes place, and the blood serum becomes saturated with free hæmoglobin, which rapidly stains the skin and sclera an intense yellow, and is excreted by the kidneys, coloring the urine, in proportion to amount, from a pale wine color to black.

Malarial hæmaglobinuria is one of the pernicious forms of malarial fever, usually following chronic malarial poisoning, due to the æstivo-autumnal parasite, and I can call to mind nothing, in the domain of practical medicine, productive of greater harm to people living in highly malarious localities, and patients suffering from malaria, than the theory of Koch and his followers, that hæmoglobinuria is due to the use of quinine.

The cycle of development of the malarial parasite takes place in the red-blood corpuscles, where they generate toxins, which cause destruction of the corpuscles, and under ordinary conditions the hæmoglobin is converted into bile pigment, and bilious, remittent fever results. But in the pernicious forms the hæmoglobin is set free in such enormous quantities that the liver can not dispose of it, and hæmoglobinuria results.

That malarial poison is a cause of nephritis, no one denies, and that a large number of the uriniferous tubules are almost totally denuded of their pavement epithelium and filled to a great extent with coagulated blood and debris by reason of the congested and inflamed condition of the kidneys, has been verified by post-mortem findings; and chemical analysis has shown that eleven-twelfths (11/12ths) of the urea which should pass out of the system through the kidneys is retained in the circulation and should relief not be speedily obtained the patient will most surely die of uremic poisoning, and Dr. Bush, of Leesburgh, Florida, reports a case in which suppression occurred after the urine had cleared up, which resulted in death.

Dr. Thayer, of Baltimore, and Dr. Forchheimer, of Cincinnati, have reported nephritis in 50 per cent of malarial cases, which were due to malaria.

Dr. Osler on one occasion said: "It was interesting to note that in the hospital there had been no single case of malarial hæmaturia which could be attributed to the use of quinine, and many physicians in the South hesitated to use quinine on account of this complication."

In opposition to the various authorities who oppose quinine are the modern text-books, and especially the recent writings of Drs. Hare and Tyson.

Then, in view of these facts from such eminent authorities, and the multiplied hundreds of reported cases in which quinine was used in continuous large doses with the happiest results, how can this dread malady be attributed to the *slightly* irritant action of quinine upon the kidneys? Dr. Moffat says "that the whole theory of the influence of quinine in causing hæmaglobinuria rests on the fact that in the majority of cases there is a history of quinine having been taken; but this fact proves nothing, when it is remembered that in tropical countries practically everybody takes quinine."

Quinine should not be condemned merely because it is an irritant to the genito-urinary tract and produces some peculiar metabolism in the circulation, but should be administered judiciously, for the fact of that morbid condition's presence is certain evidence that quinine had been neglected when first needed, and if every person requiring quinine were to take it at the first warning, and take it intelligently, there would never be another case of malarial hæmoglobinuria.

After stating the plan of treatment I almost invariably adopt in this malady, I will briefly consider the reasons I have for pursuing such a course.

When called to a case of malarial hæmoglobinuria, I proceed in the following order, being governed somewhat by existing conditions: If I find the patient with sick stomach or very nervous, which is usually the case, I administer an hypodermic of morphine and atropine, and repeat the dose as often as is necessary to control the vomiting, and without delay give three or four 5-grain doses of calomel and soda, and even more than this if required to get the liver active, the amount depending largely upon the quantity of morphine given. Give nitro-glycerine 1-100 of a grain every three hours and the following prescription:

℞ Tr. opii-camph, 4 drams.
Spts. of turpentine.
Copaiba, each 7 drams.
Spts. ether nitrosi, q. s., 4 oz.

Mix and direct. Teaspoonful every four hours.

Give fifteen grains of hydrochlorate of quinine hypodermically, and repeat the dose every six hours. Fifteen grains of quinine will dissolve readily in thirty minims of water. Instead of repeating the hypodermic dose, Warburg's pills (two grains each) every four hours, or 5-grain doses of quinine every four hours may be given by the mouth; the best results are obtained by administering it in solution, as in this form only will its prompt absorption be absolutely assured. Also, after using the first dose hypodermically, 3 or 5-grain doses of quinine, preferably the sulphate, given every two hours; the time so arranged that the last dose will be given about 2 hours before the expected paroxysm, will prove effectual.

As stated above, the morphine and atropine are to quiet the patient and check the vomiting; the latter condition may require such adjuncts as 1/10th grain doses of calomel every half hour, and a mustard plaster to the epigastrium. The calomel and soda excite the emunctories to proper activity, particularly the liver.

The nitro-glycerine stimulates the heart, and dilates the blood vessels, especially the kidneys, which are prone to occlusion by the debris.

The paregoric serves as an adjunct to quinine, acts as a sedative, and promotes cutaneous transpiration.

Turpentine is diuretic and hæmostatic, and is such an ozone or oxygen carrier that it is highly useful when quinine is given.

Copaiba is slightly diuretic, and is thought by some writers to dilate the renal vessels.

Spirits of nitrous ether by relaxing the renal vessels, is diuretic, the water being increased; it is useful when a free watery flow of urine is required to wash out the tubules and passages, and relax spasm of the renal vessels.

Quinine being destructive to the malarial plasmodia, it is the remedy par excellence in this malady, but it exerts very little toxic influence upon the parasites as long as they remain within the corpuscles, and should be administered at such a time that its maximum effect will be obtained at the time of sporulation of the parasite, which occurs with the paroxysm; and as quinine has its maximum effect about five hours after taken, and its height of elimination about the eighth hour, either of the plans of administration I have adopted will meet the above exigences.

By administering the drugs above mentioned, some of the ill effects of quinine will be obviated, for instance, it lessens the ozonizing and oxidizing function of the blood, whereas turpentine has great affinity for ozone, and will readily supply this waste; and its depressant action upon the heart will be counteracted by nitroglycerine.

The quinine is given, therefore, not in the hope of averting the pending paroxysm, but for the purpose of destroying the free, young segments upon which the succeeding paroxysms depend, and in cases where quinine had been freely given before the paroxysm immediately preceding the appearance of the hæmoglobinuria, then discontinued by its opponents, and the patients make uneventful recoveries, it is evident to my mind that the young segments were all destroyed by the quinine at the time of sporulation.

